# Developing an *In Vitro* Model to Screen Drugs for Nerve Regeneration

**DOI:** 10.1002/ar.23918

**Published:** 2018-10-17

**Authors:** MELISSA L. D. RAYNER, SIMÃO LARANJEIRA, RACHAEL E. EVANS, REBECCA J. SHIPLEY, JESS HEALY, JAMES B. PHILLIPS

**Affiliations:** ^1^ Biomaterials & Tissue Engineering UCL Eastman Dental Institute WC1X 8LD, London UK; ^2^ Department of Pharmacology UCL School of Pharmacy 29‐39 Brunswick Square WC1N 1AX, London UK; ^3^ UCL Centre for Nerve Engineering University College London WC1E 6BT, London UK; ^4^ UCL Department of Mechanical Engineering University College London WC1E 7JEK, London UK

**Keywords:** peripheral nerve, regeneration, therapies, drug discovery, tissue model

## Abstract

Peripheral nerve injuries (PNI) have a high prevalence and can be debilitating, resulting in life‐long loss or disturbance in end‐organ function, which compromises quality of life for patients. Current therapies use microsurgical approaches but there is the potential for enhancing recovery through other therapeutic modalities such as; cell‐based conduits, gene therapy and small molecules. A number of molecular targets and drugs which have the potential to improve nerve regeneration have been identified, however, there are challenges associated with moving therapies toward clinical translation. Due to the lack of detailed knowledge about the pro‐regenerative effect of potential drug treatments, there is a need for effective *in vitro* models to screen compounds to inform future pre‐clinical and clinical studies. The interaction between regenerating neurites and supporting Schwann cells is a key feature of the nerve environment, therefore, *in vitro* models that mimic this cellular association are useful tools. In this study, we have investigated various cell culture models, including simple monolayer systems and more complex 3D‐engineered co‐cultures, as models for use in PNI drug development. Anat Rec, 301:1628–1637, 2018. © 2018 The Authors. *The Anatomical Record* published by Wiley Periodicals, Inc. on behalf of American Association of Anatomists.

Damage to the peripheral nervous system can be debilitating and result in loss of motor and sensory function, coupled with poor neuron regeneration capacity (Hoke and Brushart, 2010). Despite increased understanding of the complex neuropathophysiology following such an injury, the principal clinical treatments have not changed in over 30 years (Faroni et al., 2015). Microsurgery remains the gold standard therapy, but clinical outcomes are often insufficient and the patient is unlikely to achieve complete recovery and regain function (Hoke, 2006; Geuna et al., 2009). These surgical interventions focus on bridging the injury site and do not address the complexity of cellular and molecular events occurring along the entire length of the nerve (Faroni et al., 2015). Consequently, other approaches to improve the clinical outcome need to be considered.

At present, there are no drug treatments routinely given following a PNI to improve regeneration, despite increased understanding of the specific molecular and cellular events that occur following injury. Appropriately targeted therapeutic agents have the potential to maintain neuronal viability, encourage axonal growth over larger inter‐stump gaps, improve axonal specificity to end‐organ targets, and manage neuropathic pain (Hall, 2005; Faroni et al., 2015). Therefore, there is an unmet clinical need for pharmacological therapies to improve outcomes (Martinez de Albornoz et al., 2011).

The rate of attrition in drug discovery is high with 50% of Phase III clinical trials failing and only 12% of compounds tested in humans making it onto the market (Chuang‐Stein et al., 2004; Huang et al., 2011). Pre‐clinical studies determining the efficacy and safety of compounds are required by regulatory bodies, for example the Medicines and Healthcare Products Regulatory Agency (MHRA) or the US Food and Drug Administration (FDA) before any clinical trial application (Steinmetz and Spack, 2009; Food and Drug Administration [FDA], 2015). One reason drug therapies fail to reach the market, is the lack of effective pre‐clinical models that mimic the pathophysiology and disease states in humans to test potential compounds (Huang et al., 2011). In addition, gaps in the understanding of cellular and molecular mechanisms can make it challenging to correlate the relationship between therapeutic targets and pathophysiological conditions, making it difficult to predict therapeutic outcomes (Huang et al., 2011).

The pre‐clinical stages of the drug discovery pipeline can be divided into two parts (1) *in vitro* cell culture based studies and (2) animal studies. These are vital steps and provide the raw data required to inform future clinical trials (Steinmetz and Spack, 2009; FDA, 2015). In nerve repair as in other fields, numerous models have been established to test the efficacy of compounds pre‐clinically, and increasingly a range of *in vitro* approaches are used prior to animal testing. However, most tend to use monolayer cell cultures which have limitations that may reduce their usefulness.

Despite advantages associated with their simplicity and low cost, monolayer cell culture models often fail to represent the complex 3D characteristics of living tissues (Hopkins et al., 2015; Ko and Frampton, 2016). As cells are forced to adhere to a flat stiff plastic or glass surface during monolayer culturing, they respond to spatial and mechanical cues that are not present in their natural environment, which can significantly impact their behavior; function, growth, and morphology (Ko and Frampton, 2016). The development of effective 3D *in vitro* models potentially enables researchers to bridge between results obtained in monolayer *in vitro* models and *in vivo* studies more successfully (Hopkins et al., 2015). Consequently, as 3D cell cultures advance they could help improve the pre‐clinical development process and the success of clinical trials (Ko and Frampton, 2016).

In addition, cell culture models commonly incorporate enriched monocultures of single cell types and do not consider the synergistic effect of the other cells present in nerve tissue, resulting in an unrealistic representation of the physiological environment (Ahmed et al., 2006; Kofron et al., 2009; Kraus et al., 2015; Ko and Frampton, 2016). During successful regeneration following nerve injury, Schwann cells provide support to the neurons and, therefore, this 3D cell‐to‐cell interaction is a key feature that should be recreated to build more representative *in vitro* models (Chan et al., 2014). Various 3D culture model systems have been adopted by the biomaterials and tissue engineering communities to study the influence of extracellular matrices in neurite formation and growth, but these tend not to have been developed specifically for compound screening and drug discovery (Baldwin et al., 1996; Herbert et al., 1998; Pittier et al., 2005; Ahmed et al., 2006; Bozkurt et al., 2007; Kofron et al., 2009; Kraus et al., 2015).

To maximize benefit to PNI research, new *in vitro* models need to build on and complement existing approaches while overcoming particular limitations. Animal models of PNI repair such as crush or transection and repair of a nerve in rodents are widely used to test potential treatments (Angius et al., 2012). However, while the complexity of such models echoes that of the human clinical situation and provides useful systems‐level information, this complexity can be a hindrance to understanding fundamental cellular processes and mechanisms of action. Cell culture models on the other hand permit a much greater degree of control and monitoring, enabling specific variables to be isolated and detailed cellular responses to be measured. Furthermore, cell cultures are amenable to continuous monitoring whereas cell and molecular information from animal experiments tends to be available only as a snapshot at the end‐point. The potential for use of human cells in culture models may also help to circumvent some of the concerns about differences in responses to drug treatments between species. Finally, the increased use of more sophisticated non‐animal models can help reduce, refine, and replace the use of animals in many experiments, in line with the requirements of the 3Rs in research (Russel and Burch, 1959).

One of the leading goals for drug therapies in PNI treatment is to increase the rate of regeneration of neurites as they extend through the injury site, and also through the distal nerve segment, where they are supported and guided by columns of Schwann cells that form the Bands of Büngner. A culture model that recreates this 3D neuron‐glial interaction in a reliable and reproducible manner would be a useful additional tool for testing potential drug compounds that could modulate regeneration.

Engineered neural tissue (EngNT) refers to anisotropic cellular hydrogels that have been developed for use in nerve repair to deliver Schwann cells or Schwann cell‐like therapeutic cells to mimic an autograft (Georgiou et al., 2013; Martens et al., 2014; Georgiou et al., 2015; Sanen et al., 2017; O'Rourke et al., 2018). This approach, however, also has the potential to be used as a model for pre‐clinical *in vitro* drug screening as it enables quantification of the regeneration of neurons within an aligned 3D Schwann cell‐seeded collagen gel environment. When neurons are co‐cultured with EngNT, neurite growth is supported and guided by aligned glial cells in a soft 3D extracellular matrix environment. This neurite growth *in vitro*, therefore, mimics some of the key features of the peripheral nerve environment distal to a PNI repair. By standardizing a scalable co‐culture methodology and developing robust quantitation protocols, this approach has the potential to be used as a model for drug screening.

The aim of this study was to compare monolayer and 3D EngNT co‐culture nerve tissue models as potential screening tools to enable progress toward the discovery of drug therapies for PNI. Here, the models were compared using ibuprofen as an exemplar potential drug for PNI, as it has previously demonstrated positive effects on nerve regeneration (Fu et al., 2007; Madura et al., 2011). The proposed approach will, however, be applicable to testing a wide range of other candidate drug treatments.

## METHODS

### Cell Culture

#### SCL4.1/F7 Schwann cell line

Schwann cell line SCL4.1/F7 (Health Protection Agency) cells were grown in culture medium (DMEM; Sigma‐Aldrich) supplemented with 10% heat inactivated fetal bovine serum (FBS) (Biosera) and 100 U/mL of Penicillin, 100 μg/mL of Streptomycin, in standard cell culture flasks. The cultures were maintained at sub‐confluency at 37°C with 5% CO_2_ and passaged when required.

#### PC12 neuronal cell line

PC12 cells (rat neuronal cell line, 88,022,401; Sigma‐Aldrich) were grown in suspension in culture medium (RPMI 1640; Sigma‐Aldrich) supplemented with 100 U/mL of Penicillin, 100 μg/mL of Streptomycin, (Sigma‐Aldrich), 2 mM l‐glutamine, 10% vol/vol heat‐inactivated horse serum, and 5% vol/vol fetal calf serum (Sigma‐Aldrich) in standard cell culture flasks. The cultures were maintained at sub‐confluency at 37°C with 5% CO_2_ and passaged when required.

#### NG108–15 neuronal cell line

NG108–15 cells (rat neuronal cell line, Sigma‐Aldrich) were grown in culture medium (DMEM, Sigma‐Aldrich) in standard cell culture flasks. The cultures were maintained at sub‐confluency at 37°C with 5% CO_2_ and passaged when required.

#### Dorsal root ganglion harvest and culture

Dorsal root ganglion (DRG) neurons were obtained from adult Sprague Dawley rats. The spinal column was excised from rats that were culled using a Schedule 1 method (CO_2_ asphyxiation) according to local regulations. Using an Olympus SZ40 dissecting microscope the DRGs were removed and incubated with 0.125% collagenase type IV (Sigma‐Aldrich) at 37°C for 90 min, then dissociated by trituration. Collagenase was removed by two consecutive 20 mL washes using culture medium (DMEM; Sigma‐Aldrich) supplemented with 10% heat inactivated fetal bovine serum (FBS) (Biosera) and 100 U/mL of Penicillin, 100 μg/mL of Streptomycin.

Crude DRG cell suspension was incubated in DMEM culture medium supplemented with 0.01 mM cytosine arabinoside (Sigma‐Aldrich) in a poly‐d‐lysine (Sigma‐Aldrich) coated flask at 37°C, 5% CO_2_ for 24 hr. Cells were detached using 0.25% (wt/vol) trypsin–EDTA solution and centrifuged at 400*g* for 5 min. The supernatant was discarded and the DRGs were re‐suspended in DMEM culture medium for use in experimental studies.

### Monolayer Cell Cultures

Neuronal cells (PC12, NG108‐15, and DRGs) were seeded at a density of 10,000 cells for cell lines, or cells from one DRG per coverslip, onto poly‐‐lysine (Sigma‐Aldrich, St. Louis, MO) coated coverslips (13 mm) for analysis.

### Fabrication of 3D EngNT Co‐cultures

Anisotropic cellular gels were prepared as described previously (Phillips and Brown, 2011). Briefly, 1 mL of a solution containing; 80% (vol/vol) Type I rat tail collagen (2 mg/mL in 0.6% acetic acid, First Link), 10% (vol/vol) minimum essential medium (Sigma‐Aldrich), 5.8% (vol/vol) neutralizing solution (TAP Biosystems), and 4.2% Schwann cell suspension (4 × 10^6^ SCL4.1/F7 cells per 1 mL gel) was integrated with tethering mesh at opposite ends of a rectangular mold (Dimensions 16.4 mm × 6.5 mm × 5 mm) (East et al., 2010). Cellular gels were immersed in 10 mL DMEM medium and incubated at 37°C with 5% CO_2_ for 24 hr to enable cellular self‐alignment, then stabilized using plastic compression (120 g weight for 1 min). Each stabilized aligned cellular gel was cut into four equal segments to obtain a control within each set of gels. Each gel segment was transferred to a separate well in a 24‐well plate, then 100,000 PC12 or NG108–15 cells, or DRGs in 50 μL medium were seeded on top of each segment for co‐cultures. The gels were incubated for 1 hr at 37°C to allow attachment of neuronal cells to the collagen gel, then 1 mL of culture medium (DMEM; Sigma‐Aldrich), supplemented with 10% (vol/vol) heat inactivated fetal bovine serum (FBS) (Biosera), 100 U/mL of Penicillin and 100 μg/mL of Streptomycin, was added to each well. The neurons were seeded onto the top surface of the gels and neurites extended across the horizontal plane following the aligned Schwann gels (Fig. 3C).

### Drug Treatments

Monolayer cell cultures or 3D EngNT co‐cultures were subjected to treatments of ibuprofen or GW9662 (Generon), a potent PPAR‐γ antagonist (Lezana et al., 2016), for 72 hr before being fixed with 4% paraformaldehyde (PFA) for relevant analysis. A concentrated stock solution of ibuprofen and GW9662 (10 mM) was added directly to the media in the appropriate volume to provide the required drug concentration.

### Surgical primary repair *in vivo*

All surgical procedures were performed according to the UK Animal Scientific Procedures Act 1986 after approval by the UCL Animal Welfare and Ethics Review Board. Adult male Wister rats (220–250 g) were deeply anesthetized by inhalation of isoflurane, and the left sciatic nerve was exposed at mid‐thigh level. The nerve was completely transected and the two stumps re‐connected using two 10/0 epineurial sutures (Ethicon). An osmotic pump (Alzet Model 1004) pre‐loaded with vehicle control or drug treatment (saline or ibuprofen (7 μg/day) equivalent to the *in vitro* dose) was implanted locally parallel to the nerve. The overlying muscle layers and skin were closed, and the animals were allowed to recover and maintained for 21 days.

Animals were then culled using CO_2_ asphyxiation, according to local regulations. The repaired nerves were excised under an operating microscope and immersion‐fixed in 4% PFA. Following incubation in 30% (wt/vol) sucrose and subsequent snap freezing in FSC 22 Frozen Section Media (Leica), transverse sections (10‐μm thick) were prepared from the distal stumps, at a distance 5 mm into the nerve stump from the injury site.

### Immunocytochemistry

Following fixation with 4% PFA monolayer cells or gels were washed three times with phosphate buffered saline (PBS) and permeabilized using 0.5% Triton‐X‐100 (Sigma‐Aldrich) in PBS. Washes were repeated before blocking using horse serum (1:20 in PBS; Vector Laboratories). Gels were washed before the addition of primary antibody of interest, (mouse anti‐βIII‐Tubulin 1:400 (Sigma‐Aldrich); diluted in PBS). The antibody and gel/cells were incubated overnight at 4°C. Washes were repeated before adding the corresponding secondary antibody (Dylight anti‐mouse IgG 488 1:400, (Vector Laboratories) diluted in PBS). The antibody was washed, and the sample was stored at 4°C before viewing. Omission of primary or secondary antibody was routinely used as a control.

### Immunohistochemistry

Nerve sections were washed in immunostaining buffer (PBS together with 0.2% Triton‐X (Sigma‐Aldrich), 0.002% sodium azide (Sigma‐Aldrich) and 0.25% Bovine Serum Albumin) before the addition of horse serum (1:20 in immunostaining buffer) for 45 min. The blocking serum was then removed and sections were incubated in anti‐neurofilament antisera (1:1000 in immunostaining buffer) (Neurogentec) overnight at 4°C. The sections were then washed with immunostaining buffer before addition of the secondary antibody, Dylight anti‐mouse IgG 549 (1:200) (Vector Laboratories) and incubation at room temperature for 45 min. Sections underwent a final wash with immunostaining buffer before mounting with Vectashield (Vector Laboratories).

### Image Analysis and Quantification

Fluorescence microscopy (Zeiss Axiolab A1, Axiocam Cm1) was used to capture images of neurites from five pre‐determined fields of each gel or coverslip using a ×20 lens. The neurite number in the determined fields depended on the cell type and model used (~1–12 neurites). Briefly, the positions of the pre‐determined fields on coverslips were equally spaced (625‐μm apart) in a line across the diameter at the center of the coverslip, for the gels the same method was used but the line of fields was along the edge of the construct where alignment was greatest. The length of each neurite in each field was measured using ImageJ. Following stabilization the gel acquired a thickness of 100 μm and the neurons extended predominantly in a single horizontal plane along the top surface, following the aligned Schwann cells (Phillips, 2014). This allowed analysis of neurite growth to be comparable between both the monolayer and 3D co‐cultures.

Tile scans were used to capture high‐magnification (x20) micrographs from the entire nerve cross‐section using a Zeiss LSM 710 Confocal microscope and images were analyzed using Volocity™ 6.4 (PerkinElmer) running automated image analysis protocols to determine the number of neurofilament‐immunoreactive neurites in each transverse nerve section.

### Statistical Analysis

Normality tests were conducted on all data to determine appropriate statistical tests, and one‐way analysis of variance (ANOVA) or *t*‐tests were performed, as data followed a normal distribution. A one‐way ANOVA was followed by a Tukey or Dunnett *post hoc* test. For all tests, *P < 0.05, **P < 0.01, ***P < 0.001, and ****P < 0.0001 were considered to be significant. Data are expressed as means ± SEM when described in the text.

## RESULTS

### Effect of Ibuprofen on Monolayer Cell Cultures

#### PC12 and NG108–15 cell lines

**Figure 1:**
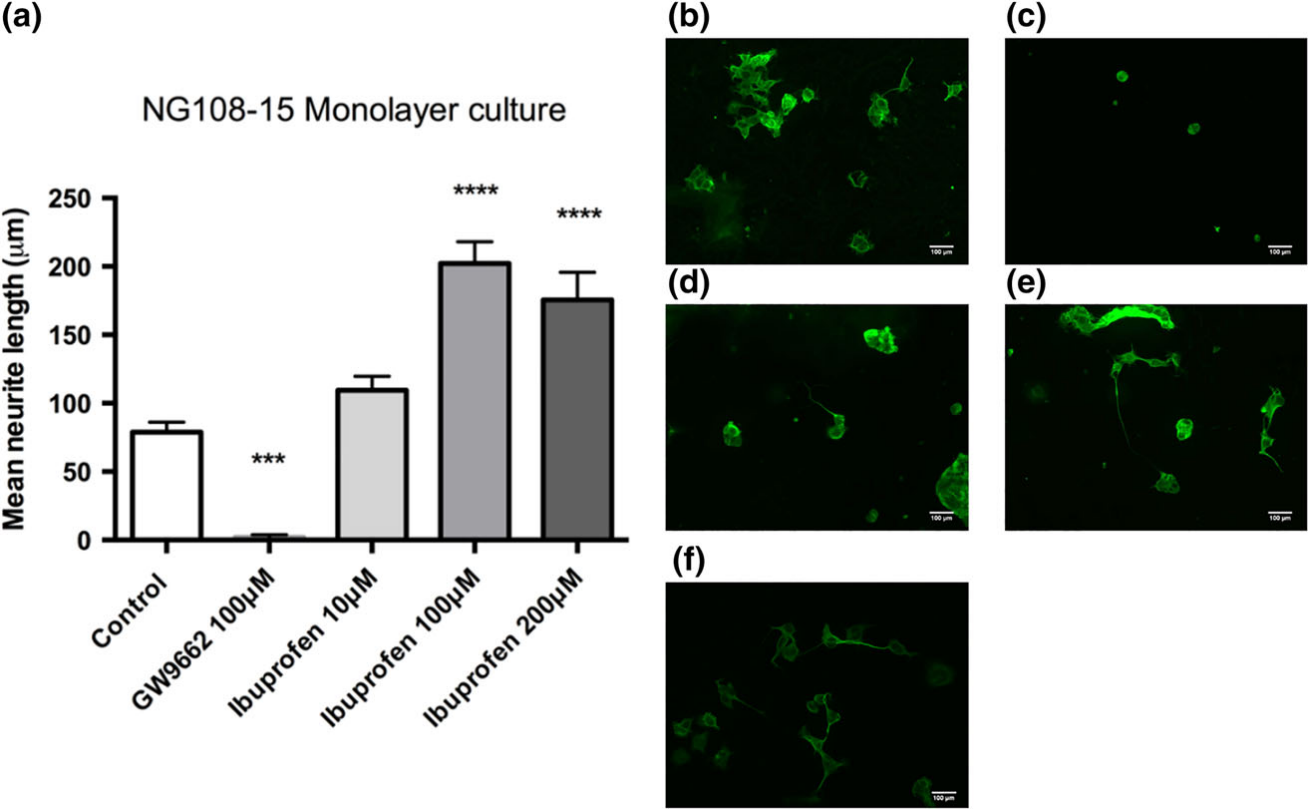
Ibuprofen induces neurite growth in monolayer NG108–15 neurons. Significant increases in neurite length were seen in the presence of 100 μM and 200 μM ibuprofen when compared to the no‐drug treatment control, after 72 hr exposure. No neurite growth was seen with the growth inhibitor GW9662 **(a).** Fluorescence micrographs of the monolayer cultures show neurite length with **(b)** no drug treatment, **(c)** 100 μM GW9662, **(d)** 10 μM ibuprofen, **(e)** 100 μM ibuprofen and **(f)** 200 μM ibuprofen. Cultures were immunostained to detect β‐III tubulin (green). N = 6, mean ± SEM for each condition. One‐way ANOVA with Tukey's *post hoc* test, ***P < 0.001 and ****P < 0.0001.

To investigate the effect of ibuprofen on isolated PC12 and NG108–15 neurons, the two cell lines were seeded onto coverslips. The cells were incubated for 72 hr with various concentrations (10, 100, and 200 μM) of ibuprofen or 100 μM of the PPAR‐γ antagonist GW9662, (Lezana et al., 2016). No neurite growth was seen with the PC12 cell line (data not shown). However, significant neurite growth was observed following treatment of NG108‐15 neurons with 10 μM (110 ± 10 μm) and 100 μM (202 ± 16 μm) ibuprofen in comparison with the no‐drug control (79 ± 7 μm) and GW9662 (Fig. 1). A higher dose of 200 μM elicited a similar effect on neurite growth to the lower dose of 100 μM.

#### Dorsal root ganglion cultures

**Figure 2:**
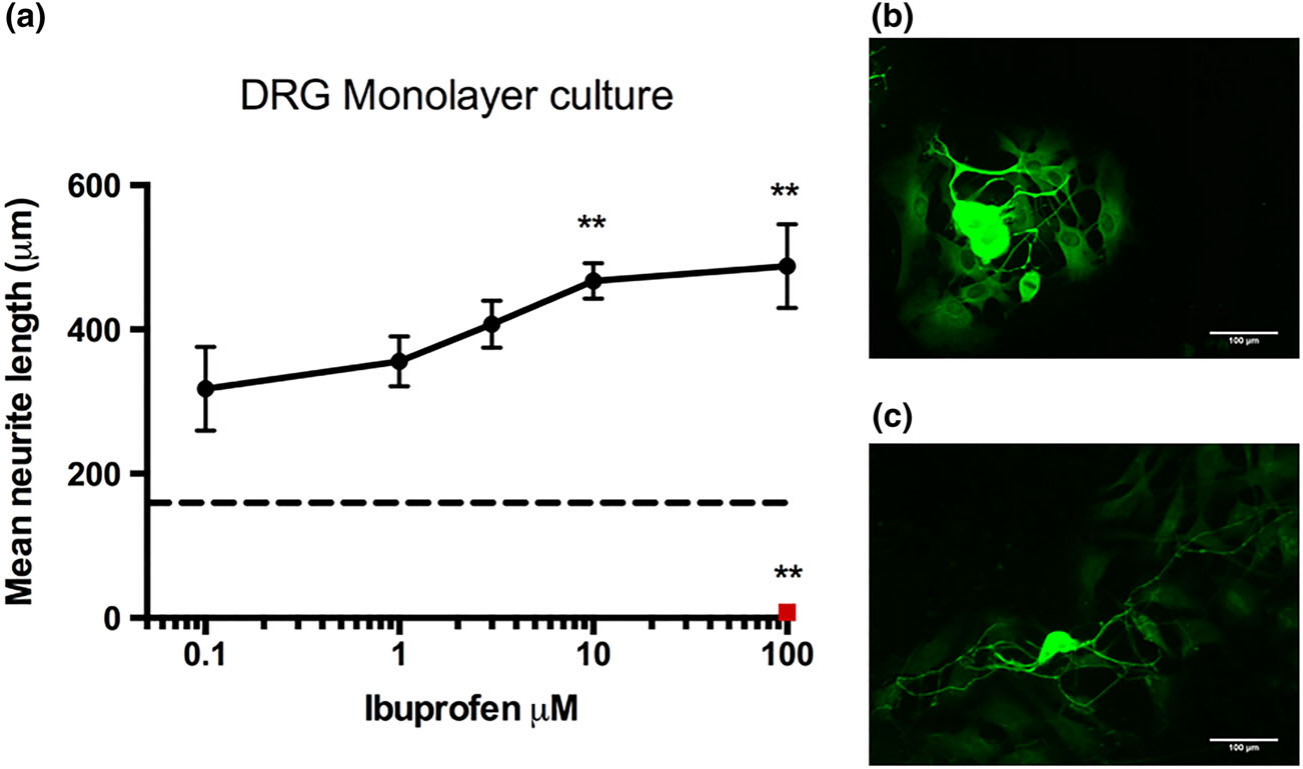
Ibuprofen induces neurite growth on monolayer crude prep DRG neurons. Significant increases in neurite length were seen with 10 μM and 100 μM doses of ibuprofen, however, all doses increased neurite growth when compared to no drug control (shown by dotted line) and 100 μM GW9662 (shown in red) after 72 hr exposure **(a)**. Fluorescence micrographs of the cultures show neurite length with no drug treatment **(b)**, and 100 μM ibuprofen **(c)**. Cultures were immunostained to detect β‐III tubulin (green). N = 6, mean ± SEM for each condition One‐way ANOVA with Dunnett's *post hoc* test, **P < 0.01.

Crude preparations of dissociated DRGs were used to determine the pro‐regenerative effects of ibuprofen on primary neurons. The monolayer neural cell cultures were coated on glass coverslips and subjected to ibuprofen treatments ranging from 0.1 to 100 μM, or 100 μM GW9662, for 72 hr. Ibuprofen treatment led to an increase in neurite length in a dose‐dependent manner with a significant increase seen with 10 μM (467 ± 25 μm) and 100 μM (488 ± 58 μm) in comparison to the no‐drug control or GW9662 treatment (Fig. 2).

### Effect of Ibuprofen in 3D EngNT Co‐cultures

#### PC12 and NG108–15 cell lines

**Figure 3:**
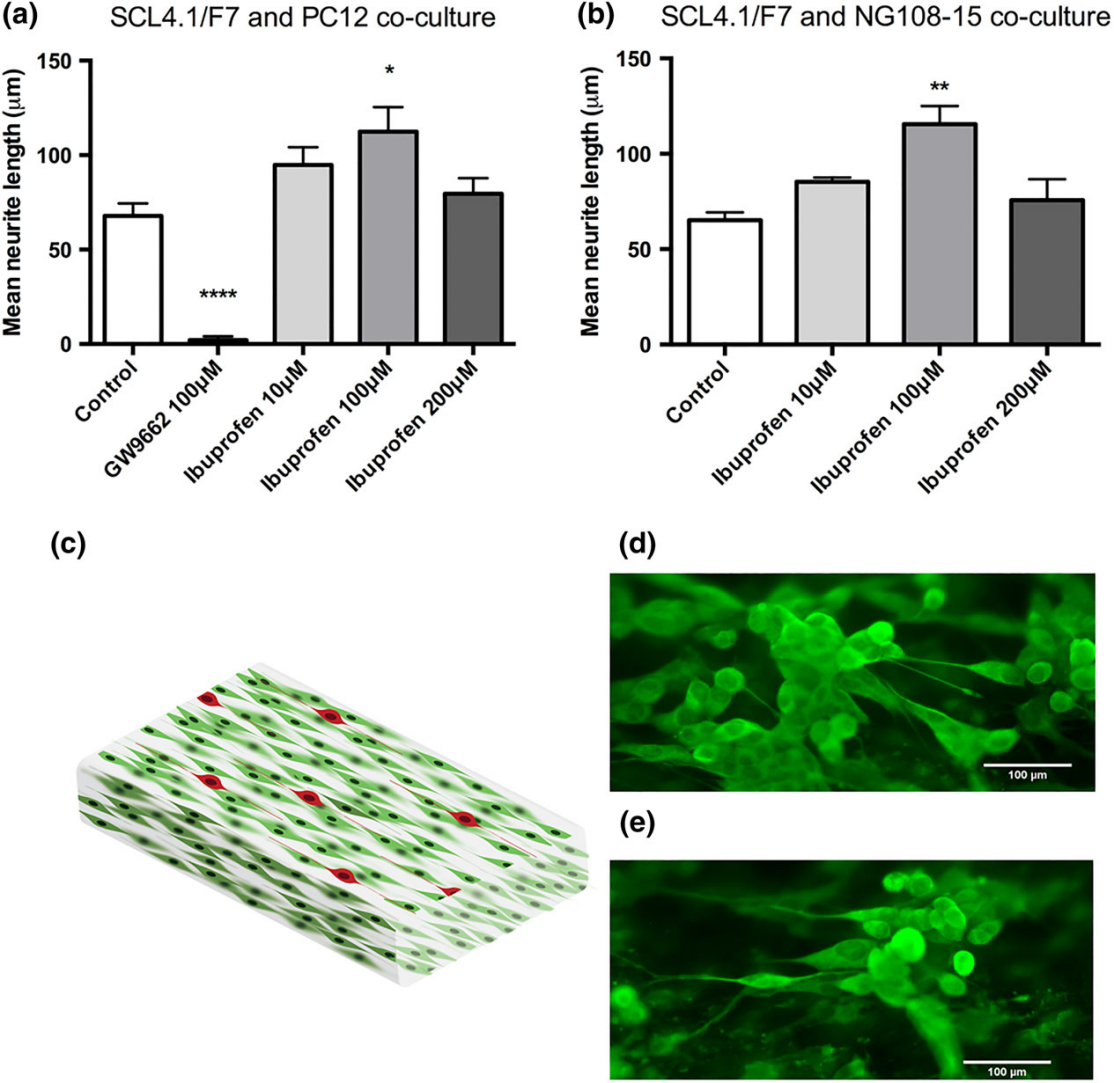
Ibuprofen increases neurite growth in the 3D EngNT co‐culture model with both cell lines; **(a)** PC12 and **(b)** NG108–15. A significant increase in growth was seen with 100 μM ibuprofen but not with 10 μM with both cell lines. Additionally, no significant increase in neurite growth was seen with a 200 μM dose. Representation of 3D EngNT containing aligned Schwann cells (green) and neurons seeding on the gel surface (red) **(c)**. Fluorescence micrographs of PC12 neuronal cells seeded on the gels without **(d)** and with **(e)** 100 μM drug treatment. Cultures were immunostained to detect β‐III tubulin (green). N = 6 of PC12 co‐culture and N = 3 of NG108–15 co‐culture gels, mean ± SEM for each condition. One‐way ANOVA with Dunnett's *post hoc* test, *P < 0.05, **P < 0.01, and ****P < 0.0001.

Three‐dimensional EngNT co‐culture models were used to investigate the capacity of ibuprofen to modulate neurite outgrowth in two neuronal cell lines; PC12 and NG108–15. Ibuprofen was found to induce neurite extension in both cell lines in a similar dose‐dependent manner at concentrations of 10 μM (PC12: 101 ± 10 μm, NG108: 85 ± 2 μm) and 100 μM (PC12: 112 ± 13 μm, NG108: 116 ± 10 μm) ibuprofen, however, minimal increase in neurite length was seen with a dose of 200 μM ibuprofen (Fig. 3). An increase in comparison to the control and antagonist GW9662 was observed at concentrations of 10 and 100 μM.

#### Dorsal root ganglion neurons

**Figure 4:**
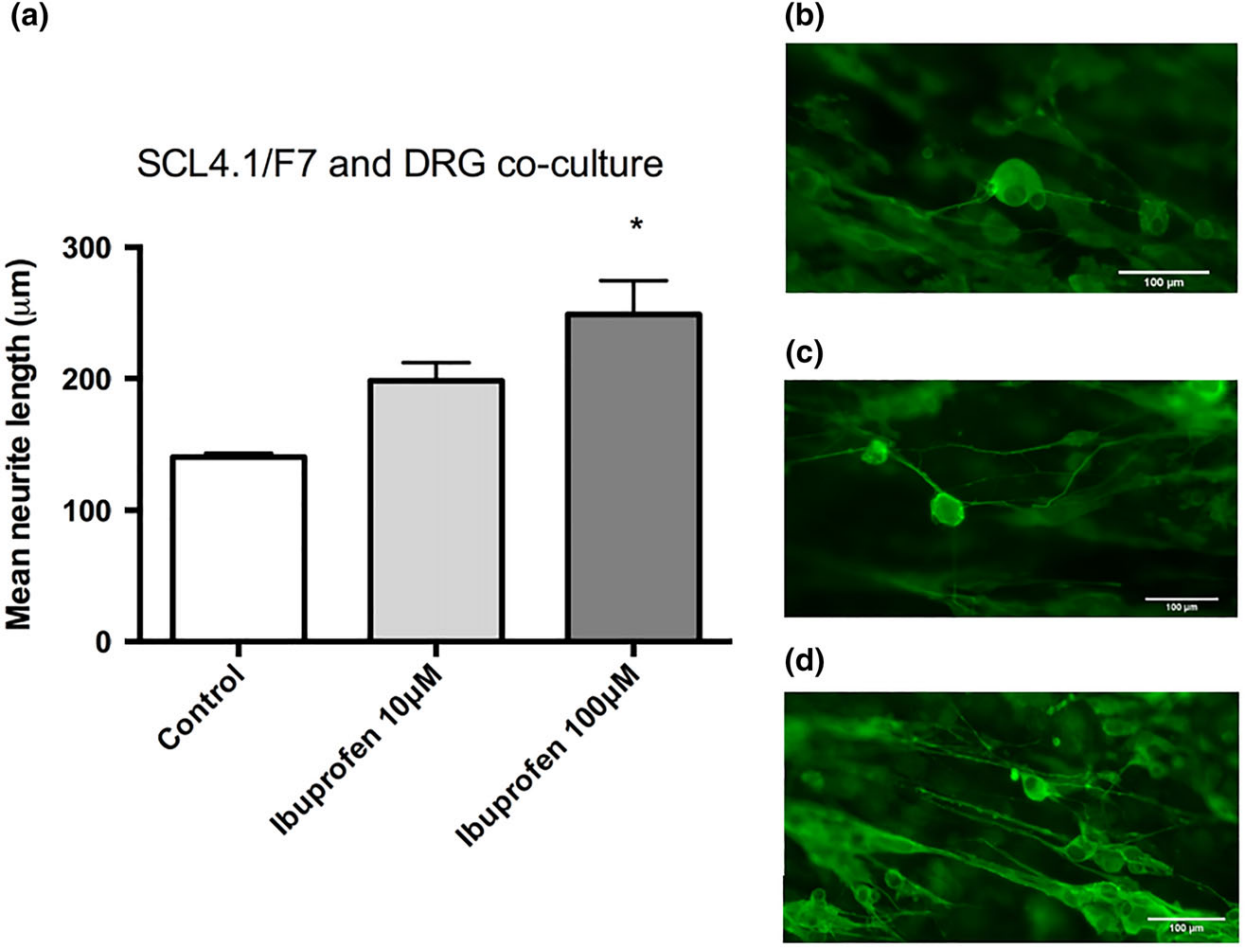
Ibuprofen induces neurite growth in the 3D EngNT co‐culture model with DRGs **(a)**. A significant increase was seen with a dose of 100 μM ibuprofen but not 10 μM. Fluorescence micrographs of DRG neurons on EngNT gels without **(b)** and with **(c)** 10 μM drug treatment and **(d)** 100 μM drug treatment. Cultures were immunostained to detect β‐III tubulin (green). N = 6, mean ± SEM for each condition. One‐way ANOVA with Dunnett's *post hoc* test, *P < 0.05.

Three‐dimensional EngNT co‐culture models were fabricated using DRG primary neuronal cells to determine the effect of ibuprofen on neurite outgrowth. When treating the DRGs with ibuprofen, a dose response similar to that with PC12 and NG108–15 cell lines was observed. At concentrations of 10 μM (198 ± 14 μm) and 100 μM (249 ± 26 μm), a significant increase in neurite length in comparison to the control (140 ± 3 μm) was seen (Fig. 4), however, the mean neurite growth was longer with DRGs.

#### *Regenerative capacity of ibuprofen* in vivo

**Figure 5:**
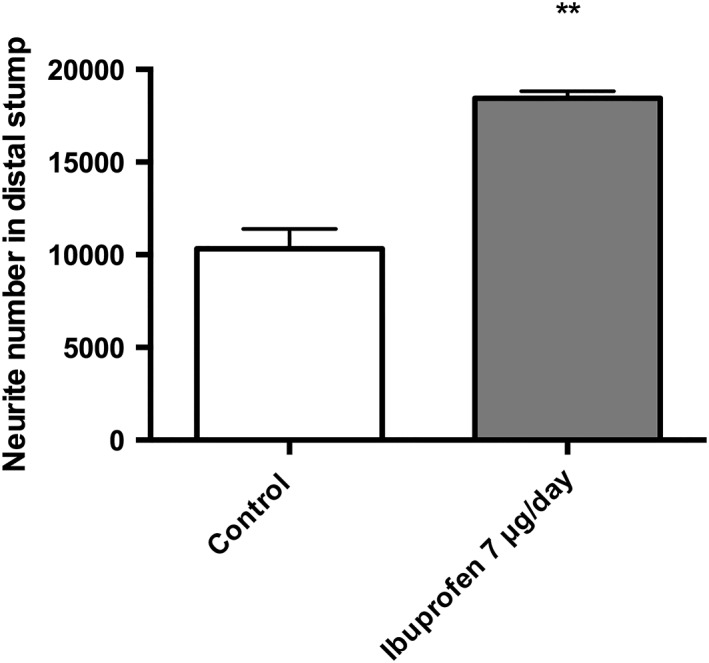
Ibuprofen increases the number of neurites detected in the distal stump 5 mm from the injury site following a local dose of 7 μg/day ibuprofen for 21 days. N = 3 animals per group. Mean ± SEM for each condition. *T*‐test, **P < 0.01.

Transverse sciatic nerve sections were analyzed for axon number. Results demonstrated that a dose of 7 μg/day ibuprofen increased the number of axons in the distal stump in comparison to no drug treatment (Fig. 5). Furthermore, the number of axons quantified in the distal stump of the ibuprofen group exceeded the axon number in the corresponding proximal stump of the same animal (data not shown).

## DISCUSSION

There is a growing interest in the development of *in vitro* models that can accurately simulate the natural regeneration process of neurons following a PNI. Monolayer *in vitro* systems have been developed by co‐culturing neurons with Schwann cells to study the influence of cell‐to‐cell interactions (Clarke et al., 2011), however, more complex 3D co‐culture systems have been developed to mimic the natural nerve environment more closely such as; explant cultures (Yip et al., 1998; Miller et al., 2001; Vikman et al., 2001; Lesuisse and Martin, 2002) self‐assembled aggregate cultures (Knoops et al., 1991; Becq et al., 1999; Jerregard, 2001; Lillesaar et al., 2001; Ruscheweyh and Sandkuhler, 2001; Edoff and Jerregard, 2002; Lee et al., 2002), and scaffold based cell cultures (Borkenhagen et al., 1998; Dubey et al., 1999; Backstrom et al., 2000; Balgude et al., 2001; Yu and Bellamkonda, 2001; Georgiou et al., 2013).

Regardless there is still an unmet requirement for a model to screen the effect of drug therapies on neuron regeneration. This study investigated the effectiveness of different culture systems in terms of suitability for use in PNI drug development, by investigating the effect of ibuprofen on the length of neurites in the culture systems in comparison with an *in vivo* study. Overall, ibuprofen increased neurite length in the models regardless of the type of neurons and the complexity of the culture environment. This was consistent with the *in vivo* study, and also mimicked effects reported following treatment with ibuprofen in previous studies.

These studies have shown neurite growth enhancement in primary neuronal cell cultures following ibuprofen treatment in a dose‐dependent manner (Fu et al., 2007; Wang et al., 2009; Dill et al., 2010). The effect of ibuprofen on neurite growth has also been demonstrated in rat models of both the central and peripheral nervous system, with axonal sprouting induced by ibuprofen treatment following a contusion injury (Wang et al., 2009), the promotion of axonal growth following spinal cord lesions (Fu et al., 2007), and improved axonal areas and myelin in peripheral nerve repair (Madura et al., 2011). Adding to these earlier findings, this is the first report showing a significant increase in neurite number in the distal stump induced by ibuprofen treatment following a primary repair in a rat sciatic nerve.

Similar patterns of dose–response were achieved following ibuprofen treatment when using either the PC12 or NG108–15 neuronal cell lines or primary neurons in the 3D EngNT co‐culture model. With all cell types, the optimal dose was 100 μM ibuprofen which increased growth by ~60%–70%. In contrast, in the monolayer cultures no growth was seen with the PC12 neuronal cell line, indicating that the 3D EngNT co‐culture environment is required in order for that cell line to extend neurites in these studies.

Nonetheless, the results observed from the monolayer NG108–15 and DRG cultures still demonstrated extensive neurite elongation following ibuprofen treatment. Interestingly, the extent of growth in these cultures was different to that seen in the 3D system. Even though the optimal dose remains as 100 μM ibuprofen, the growth increase in response to the drug exceeds 100%, considerably higher than that seen in the 3D system. Also the 200 μM dose in the NG108–15 monolayer culture elicited an increase in neurite length in comparison to the control which was not observed in the 3D‐engineered co‐culture.

**Figure 6:**
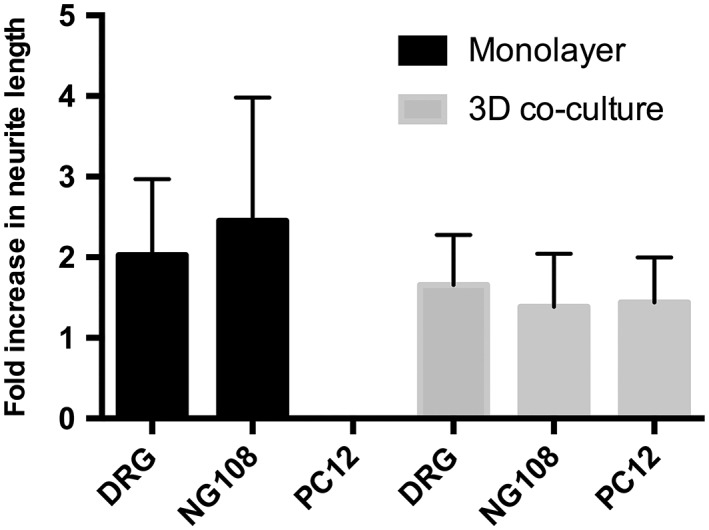
The data sets for the 100 μM dose of ibuprofen in the various different *in vitro* models were normalized to their respective controls and presented as fold changes over the no drug treatment condition. Neurite growth increase was seen in all of the *in vitro* models except the monolayer PC12 cultures where no growth was seen. N = 6 (N = 3 for NG108–15 3D co‐culture). Mean ± SD.

To progress toward the development of a model to be used as a screening tool for PNI therapies, a metric was devised to compare the different *in vitro* models and to determine which one best predicted the effect seen *in vivo*. The *in vivo* model demonstrated a twofold increase in neurite number following 7 μg/day of ibuprofen administered locally for 21 days. The dose administered *in vivo* was approximately equivalent to the 100 μM dose the cells were subjected to *in vitro*. Therefore, the data sets for this dose were analyzed from each culture model, by normalizing them to their controls. This analysis showed that the monolayer models had marked variability in neurite length. Although the DRG and NG108–15 monolayer cultures had comparable fold increases, they had a large standard deviation (e.g., ±60% in the NG108–15 culture) (Fig. 6). Conversely, the 3D models were more consistent, with a standard deviation of ±40% independent of the cell type used. Furthermore, the 3D EngNT co‐culture with DRG neurons demonstrated a fold increase in neurite length that is most comparable to the *in vivo* model.

Cell choice is an important consideration for *in vitro* experiments and there is invariably a trade‐off between the ready availability, standardization, and reproducibility of immortalized cell lines (e.g., NG108–15, PC12, SCL4.1/F7) and the use of primary cells which behave more naturally but have additional limitations in terms of source and variability. Species differences may also be a consideration depending on the purpose of the experiment (as a precursor to animal studies then animal cells may be more appropriate, but in turn they may be less appropriate for modeling human physiology). Comparable effects were seen between cell lines and primary neurons following ibuprofen treatment in this study, indicating that the cell lines provided a valid prediction of the effects of drugs without the need for primary cells in this instance. Further development to ensure the model is reproducible in other laboratories, with defined intra and inter observer reliability and intra class correlation, will be required to facilitate scalable manufacture and subsequent widespread adoption.

## CONCLUSION

The synergistic activity of neurons and Schwann cells plays a key role in nerve regeneration; therefore, it is desirable to recreate this key cell‐to‐cell interaction in a model when evaluating the effects of drug therapies. Using a tool that is more biologically comparable than a monolayer culture of isolated cells may provide pertinent data on drug safety and efficacy to inform subsequent animal and clinical studies. This 3D EngNT co‐culture model system allows the exploration of key regenerative events such as neurite formation and optimal dosing regimens and could also be used to understand the mechanism of action of drug candidates.

Using 3D co‐culture systems increases complexity and can introduce time restraints and restrictions to the number of samples (Ko and Frampton, 2016), however, our analysis indicates that the 3D EngNT co‐culture systems are predictive of ibuprofen response *in vivo*, and also more predictable in terms of outcome measures than their monolayer counterparts.

In conclusion, we have taken a step closer to generating a model that represents the complex environment of PNI, providing for the first time a 3D co‐culture model of PNI developed specifically for drug discovery and development. The model enables medium throughput drug screening with results that are predictive of the neuro‐therapeutic outcome.
